# Supplementary material for: Mechanisms governing the pioneering and redistribution capabilities of the non-classical pioneer PU.1
Source: Nat Commun. 2020 Jan 21;11:402. doi: 10.1038/s41467-019-13960-2 (PMC6972792; doi:10.1038/s41467-019-13960-2)
Supplement: Supplementary file 7 — Source data [file 41467_2019_13960_MOESM7_ESM.zip › Source_Data/Figure5/Figure5A_MotifScanOutput/homerResults/motif12.similar.html]

motif12

## Information for motif12

A
G
T
C
C
A
G
T
A
C
T
G
A
C
G
T
C
T
G
A
C
T
A
G
A
C
T
G
A
G
T
C
A
C
G
T
A
G
T
C
A
G
T
C
C
T
G
A
A
G
T
C
A
G
T
C
  
Reverse Opposite:  

A
C
T
G
C
T
A
G
G
A
C
T
C
T
A
G
A
C
T
G
C
G
T
A
C
T
A
G
G
T
A
C
A
G
T
C
A
G
C
T
G
T
C
A
G
T
A
C
G
T
C
A
C
T
A
G
  

|  |  |
| --- | --- |
| p-value: | 1e-55 |
| log p-value: | -1.288e+02 |
| Information Content per bp: | 1.853 |
| Number of Target Sequences with motif | 87.0 |
| Percentage of Target Sequences with motif | 2.88% |
| Number of Background Sequences with motif | 90.7 |
| Percentage of Background Sequences with motif | 0.20% |
| Average Position of motif in Targets | 154.0 +/- 96.6bp |
| Average Position of motif in Background | 213.1 +/- 138.7bp |
| Strand Bias (log2 ratio + to - strand density) | 0.2 |
| Multiplicity (# of sites on avg that occur together) | 1.30 |
| Motif File: | file (matrix) reverse opposite |

### Similar de novo motifs found

|  |  |  |  |  |  |  |  |
| --- | --- | --- | --- | --- | --- | --- | --- |
| Rank | Match Score | Redundant Motif | P-value | log P-value | % of Targets | % of Background | Motif file |
| 1 | 0.866 | C A T G G C A T C T A G A C T G T C G A C A T G T G A C G A T C G A C T G T C A T A G C | 1e-42 | -96.920227 | 2.42% | 0.21% | motif file (matrix) |
| 2 | 0.807 | A C T G A C T G A G T C A C G T A G T C A G T C C T G A A G T C | 1e-25 | -59.177298 | 5.84% | 2.25% | motif file (matrix) |
